# Mechanosensory Hairs and Hair-like Structures in the Animal Kingdom: Specializations and Shared Functions Serve to Inspire Technology Applications

**DOI:** 10.3390/s21196375

**Published:** 2021-09-24

**Authors:** Brittney L. Boublil, Clarice Anna Diebold, Cynthia F. Moss

**Affiliations:** Department of Psychological and Brain Sciences, Johns Hopkins University, 3400 N Charles St., Baltimore, MD 21218, USA; bboubli1@jh.edu (B.L.B.); clarice.diebold@jhu.edu (C.A.D.)

**Keywords:** sensory hairs, mechanosensation, bioinspired sensors

## Abstract

Biological mechanosensation has been a source of inspiration for advancements in artificial sensory systems. Animals rely on sensory feedback to guide and adapt their behaviors and are equipped with a wide variety of sensors that carry stimulus information from the environment. Hair and hair-like sensors have evolved to support survival behaviors in different ecological niches. Here, we review the diversity of biological hair and hair-like sensors across the animal kingdom and their roles in behaviors, such as locomotion, exploration, navigation, and feeding, which point to shared functional properties of hair and hair-like structures among invertebrates and vertebrates. By reviewing research on the role of biological hair and hair-like sensors in diverse species, we aim to highlight biological sensors that could inspire the engineering community and contribute to the advancement of mechanosensing in artificial systems, such as robotics.

## 1. Introduction

Across the animal kingdom, organisms have evolved specialized sensory systems to contend with complex environments and respond to biologically relevant stimuli. Sensory systems must often quickly and accurately encode stimuli to guide natural behaviors. Biological sensors often reveal high sensitivity, enable behavioral flexibility, and operate with energetic efficiency. Current artificial implementation of hair-like sensors falls short of biological counterparts. Wider knowledge of the mechanosensory organs that enable diverse behaviors across animal taxa can inspire technological advances in the development of new artificial sensors [1,2,3,4,5].

Here, we focus on the morphology, anatomical location, and proposed functions of mechanosensory hairs in vertebrate and invertebrate species. While important knowledge comes from careful measurements of the physical properties of mechanosensory hairs, and some excellent research has been done in the area [6,7,8,9], we only touch upon such measurements here, as published work is too limited to review in a comparative context. Instead, we have curated a selection of sensory hairs and hair-like structures across the animal kingdom that may provide insight or inspiration to future applications of artificial sensors.

Sensory hairs have arisen throughout the animal kingdom to enable rapid and finely tuned mechanosensory processing [10]. From deep oceans to dense forests, animals encounter both complex and dynamic environments and stimuli, such as fluid turbulence and erratically moving prey. While the general architecture of many mechanosensory hairs has been highly conserved [11], animals have evolved species-specific specializations in the use of hairs to support a multitude of complex behaviors. Hairs and hair-like structures can vary in function and sensitivity, based on details of their morphology, specialized receptors, and location on the animal’s body, all of which contribute to the rich behavioral repertoires these structures support.

**Figure 1:**
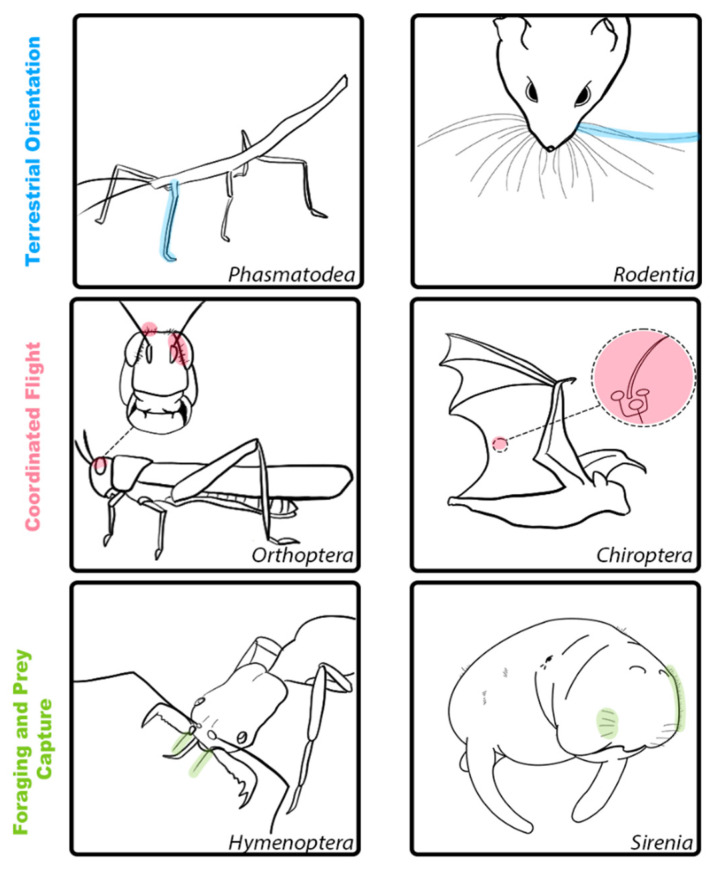
Illustrations of hair and hair-like structures with shared functional properties for select invertebrate and vertebrate species. Top row (blue): Examples of hair and hair-like structures for terrestrial locomotion in Phasmatodea (**left**) and Rodentia (**right**). These structures are involved in orientation and self-guided motion to support coordinated movement. Middle row (red): Examples of hair and hair-like structures for coordinated flight in Orthoptera (**left**) and Chiroptera (**right**). These structures detect airflow and support flight control. Bottom row (green): Examples of hair and hair-like structures for foraging and prey capture in Hymenoptera (**left**) and Sirenia (**right**). These structures are adapted to allow for species-specific foraging behaviors.

The behaviors supported by mechanosensory hairs and hair-like structures are shaped by an animal’s natural history, ecology, and niche. We have highlighted a selection of examples of specialized hair and hair-like structures in animals that provide sensory input used for the coordination of locomotion on substrates, in water and in air, the exploration of objects in their environments, navigation, and specialized foraging and prey capture behaviors (see Figure 1 and Table 1). While many mechanosensory hairs are multifunctional and carry information used to guide a variety of behaviors, we have selected a handful of illustrative examples that highlight the vast potential for these remarkable sensory structures. Further, we point to shared functions of different mechanosensory hairs and hair-like structures found in vertebrate and invertebrate species and suggest ways this knowledge can be applied to new technological advances in robotic sensing.

## 2. Mechanosensory Feedback for Coordinated Locomotion

**Figure 2:**
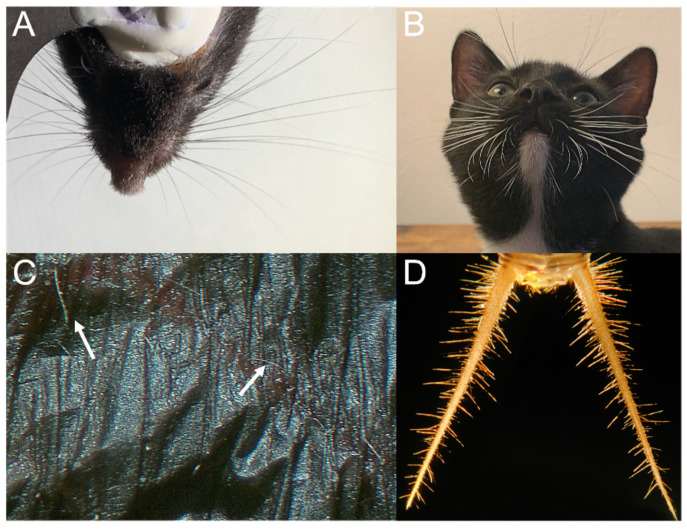
Hairs and hair-like structures aid in coordinated movement, navigation, and exploration. *Top row* (**A**,**B**) shows facial vibrissae that aid in orienting and detection. (**A**) Facial whiskers of adult mouse (*Mus musculus*, C57b6; photo credit C.A.D.). (**B**) Facial whiskers of juvenile cat (*Felis catus*; photo credit C.A.D.). *Bottom row* (**C**,**D**) shows sensory hairs that aid in airflow detection and flight control. (**C**) The wing hairs of an adult male big brown bat (*Eptesicus fuscus*; photo credit C.A.D.). *(***D**) The cerci of adult female cricket (*Acheta domesicus*; photo credit [30]).

Mechanosensory hairs and hair-like structures have evolved in diverse species to carry information for the coordination of movements for locomotion. Specifically, mechanosensory feedback provides proprioceptive information for locomotion using specialized hair and hair-like structures (see Figure 2). The coordination of self-movement is a fundamental building block that other more complex behaviors (i.e., prey capture and foraging) rely upon. Locomotion in many organisms is enabled by specialized sensory hair structures that provide key sensory feedback to coordinate the motion of limbs.

***Terrestrial invertebrates (Orthoptera, Diptera).*** Many insects, including stick insects and locusts, rely on proprioceptive input to coordinate movements like walking. Hair plates are specialized proprioceptors composed of clusters of tactile hairs, with each individual sensillum innervated by a sensory neuron [31,32]. These sensory neurons can adapt either slowly to maintained displacement or rapidly to transient hair movements [13,32,33]. Hair plates are often located in the folds of cuticles where they are displaced during joint movements, providing proprioceptive information for movement [31]. This sensorimotor feedback is essential for many insects to coordinate walking movements. For instance, ablating hair plates located on the legs of many insects lead to uncoordinated movement and overstepping, where the back legs collide with front legs, suggesting the hair plates act as a limit detector [13,33,34]. 

Recent technological innovations have provided further insight into how proprioceptive feedback can be vital for coordinated motion, revealing the importance of central pattern generating network (CPG) activity in some insects, such as stick insects and locusts, as well as inter- and intralimb sensory feedback, and/or rhythmic central networks synchronize motor patterns [35,36,37]. Recent work in locusts has described the importance of the subeosophageal ganglion (SEG) in activating leg CPGs and coordinating coupling across legs, contributing to the regulation of insect leg motion quickly and effectively [38]. Further, while there is tight coordination in the CPGs for leg movement in these insects that enables the highly stereotyped walking movements, there are also more flexible components of each segment that has its own ganglion, which can override the synchronized CPGs across segments [39]. This allows behavioral flexibility and quick responses to the environment while still enabling energetically efficient stereotyped walking movements.

In addition to providing proprioceptive feedback to coordinate locomotion, hair-like structures can also aid in generating the necessary friction for movement itself. For many insects, including flies, hairy attachment pads are critical for creating the friction necessary to maintain attachment to a surface [40,41]. Flies have pulvilli, adhesive pads covered by setae, which have specialized ultrastructures to aid in the attachment and detachment of the fly to surfaces. Some setae on the distal part of the pulvillus secrete adhesive substances close to the contact area and the seta tip, while setae on the basal part of the pulvillus do not have a secretion mechanism [12]. These two ultrastructures on the distal and basal parts of the pulvillus aid in the fly’s ability to attach to various surfaces. Hairs enabling travel along surfaces offer important advantages to many invertebrates that have evolved to operate in diverse environments. Organisms, such as caterpillars, rely on sensory input to coordinate the movement of their thoracic legs and prolegs to crawl and grip onto surfaces [42]. For many species, rows of directionally sensitive stiff sensory hairs on the lateral distal edge of the proleg project to the segmental ganglia, which can directly control motor neurons for the coordination of leg movement [42,43]. Insects must often navigate surfaces with either horizontal orientation or little frictional support to adhere to, and specialized hair structures enable many organisms to occupy ecological spaces otherwise inaccessible.

***Flying invertebrates (Orthoptera, Hymenoptera, Blattodea).*** Generally, the deflection of tactile hairs opens mechanotransduction channels that activate sensory signaling. Tactile hairs can be directionally sensitive, as well as sensitive to changes in velocity, triggering an increase in spiking activity when deflected [44]. Some insects have hairs that are specialized to detect airflow and can be deflected by slight changes in air motion, resulting in mechanosensory stimulation of receptor cells under the hair base [45]. The ability to detect airflow during flight is critical for producing rapid motor responses, particularly under windy conditions. 

Many insects, including locusts, are covered with trichoid sensilla, which can detect deflections produced through contact with objects in the environment or airflow, as described above. Some trichoid sensilla are located on the head capsule and are termed cephalic trichoid sensilla. Cephalic trichoid sensilla have explicitly been shown to be involved in flight control [8,20]. When tethered locusts were exposed to jets of air, hairs on the frons and vertex were stimulated, and flight movements were induced [8]. When the air jets were removed, flight behavior ceased. Further, when hairs were covered with cellulose paint, sustained flight could no longer be induced by airflow stimulation. These experiments showed that stimulation of airflow detectors on the heads of locusts were sufficient to induce and maintain flight. Interestingly, static mechanical stimulation of the same hairs (as opposed to the dynamic deflection from airflow) was not sufficient to induce flight [20,46,47], indicating that airflow is necessary to elicit a behavioral response. 

Specialized hairs work in concert with motor systems in the locust to coordinate flight by detecting properties of airflow. Weis-Fogh [8] first observed that locusts oriented towards the direction of airflow stimuli, suggesting that cephalic trichoid sensilla have directional tuning. When locusts detect a change in the angle of wind from deflections of their cephalic trichoid sensilla, this evokes a rudder-like movement that stabilizes and adapts relative to the magnitude of the change in the wind angle [48]. Locusts also orient their abdomen relative to changes in wind velocity as a potential response to avoid stall [49]. In addition to locusts, trichoid sensilla on the compound eyes of honeybees have been implicated in correcting for wind drift [20,50]. Trichoid sensilla are important for detecting airflow patterns and changes to elicit rapid motor adjustments that maintain and coordinate flight behavior. 

Some orthopterans, like crickets, have a cercal sensory structure, which responds to sound, vibration, and airflow [21,22,51,52]. This structure consists of a pair of appendages on the rear abdomen of the orthopteran. These appendages are covered with 1000–2000 filiform receptor hairs whose movement innervates mechanosensory afferent neurons and projection interneurons [21,22]. The cercal system in orthopterans is highly directional [52], with a hinge-like cuticle structure at the base of the hair [53]. Cerci are key for detecting changes in an environment to allow for rapid behavioral reactions, as seen in escape responses in locusts [51]. The cercal system is also implicated in maintaining control during flight. For example, in cockroaches, ablation of one cercus causes asymmetrical flight [54], suggesting bilateral cerci provide sensory feedback necessary to coordinate normal flight behavior.

***Flying vertebrates (Chiroptera).*** Bats are the only mammals capable of powered flight. Unlike other mammals in the animal kingdom [55,56,57], bats lack glabrous (i.e., hairless) skin [58]. In addition to the fur or pelage hair found covering most of the bat’s body, they also have hairs on their wings, tail, rump, and feet [59]. Kang and Reep [59] examined postcranial hairs on 66 species of bats and hypothesized that these were sensory hairs based on their structure and placement. Furthermore, they suggested that bat sensory hairs play different roles depending on their placement, as well as life history traits of each species, such as roost type, size of roosting group, and diet. They found that the shape, length, and thickness of postcranial hairs differed from those of pelage hairs, and they posited that bodily placement of sensory hairs is related to function. For instance, hairs on the tail membrane were thought to contribute to foraging behaviors and landing, whereas toe hairs were suspected to function mainly as a tool for grooming. The functions of these different types of postcranial hairs in bats are not fully understood. It is noteworthy that hairs on the wings of bats were identified over 100 years ago [60], and many decades later have been implicated in airflow sensing for flight control [58,61]. 

The membrane of bat wings is sparsely lined with microscopic hairs, which are associated with a variety of tactile receptors, including lanceolate receptors and Merkel cell neurite complexes [9,62]. It was found that two different species of bats, *Eptesicus fuscus* (big brown bat) and *Carollia perspicillata* (short-tailed fruit bat), altered their flight behavior in an obstacle avoidance task following wing hair depilation. They found that *E. fuscus* and *C. perspicillata* made wider turns around obstacles and increased their flight speed after depilation, respectively [58]. These findings suggest that wing hairs act as airflow sensors that prevent stall [9,58].

Furthermore, extracellular recordings in bat primary somatosensory cortex (S1) show neural responses to light touch and air puff stimulation of the wing; S1 responses to air puffs showed directional tuning, with a predominance of neurons responding selectively to reverse airflow [58]. Further, both air puff and tactile stimulation activated overlapping regions in S1 of the big brown bat [62]. The firing rate of S1 neurons in response to air puff stimulation diminished after wing hair depilation but showed no decline in response magnitude to light touch stimulation in the same receptive field [58], supporting the hypothesis that hairs on the wings of bats function as airflow sensors.

***Shared functional properties: Coordination of locomotion.*** Across vastly different taxa, many organisms rely on rapid integration of mechanosensory signals to coordinate species-specific movements that operate on behaviorally relevant scales. For small organisms like insects, proprioception aids in the coordination of limb movements [32] (Figure 2). Proprioceptive feedback from hair plates near the legs proves essential to locomotion in many species of insects [31]. Small insects, like flies and caterpillars, rely on strong adhesive gripping to navigate difficult terrain and adhere to vertical surfaces, which is enabled through specialized hair pads [40,41,42]. These specialized structures in insects aid in coordinating locomotion that enables organisms to inhabit challenging environments and occupy diverse ecological niches. The ability to adhere to angled surfaces and remain in place opens access to environments that are otherwise inaccessible.

In addition to locomotion on surfaces and substrates, sensory hairs also play an important role in supporting flight behaviors in insects and bats. These sensors can effectively and rapidly detect changes in airflow patterns arising from the environment (i.e., changes in weather and wind conditions) and enable coordinated flight and movement. The specific placement and structure of hairs allows for directional selectivity, allowing not just for the detection of change but also a better sense for where changes in airflow are occurring and allow the animal to quickly determine how best to respond to these changes behaviorally. Locusts have microscopic sensory hairs on their head capsules that provide information about wind speed and direction that can evoke behavioral responses to maintain flight [8,20]. Similarly, bats are equipped with microscopic hairs located on their wings, which serve as airflow sensors that signal unsteady conditions and prevent stall [9,58]. These mammals employ these hairs to fly effectively even in cluttered and complex environments, coordinating behavioral modifications relative to the navigational task.

Despite morphological and physiological differences in mechanosensory hairs across taxa, they serve common functions in providing feedback to guide locomotion. Future research on the mechanical and neurobiological mechanisms that enable high sensitivity and directionality of mechanosensory hairs guiding locomotion will enable greater agility of artificial systems equipped with sensors to travel on land or air.

## 3. Navigation and Exploration

By taking in sensory information from their surroundings, animals are able to navigate, find mates, forage, and evade predation. In many species, mechanosensory hairs contribute to the detection and exploration of objects in the environment, from obstacles and terrain to predators and prey. Here, we have selected examples from the animal kingdom that highlight the use of sensory hairs to navigate diverse habitats and to detect and discriminate the objects they encounter.

***Aquatic invertebrates (Decapoda).*** Crustaceans are arthropods that have developed specialized sensory hairs to detect changes in water flow in their environments. Water disturbances produced by other animals in a fluid environment cause flow patterns that can be detected by mechanoreceptors to enable rapid behavioral responses. These hydrodynamic cues are vital for detecting the presence of a predator, mate, or even a potential meal. While mechanosensory hairs operating in air and water serve similar functions, the comparatively high density and small kinematic viscosity of water [63] have placed evolutionary pressures on aquatic animals.

Some crustaceans, such as crayfish, are crepuscular and use non-visual cues to navigate and orient effectively. Many species rely on tactile input from the antennae to detect changes in the environment as they search for prey. Antennae can consist of short proximal segments that support multi-segmented flagellum [64]. Two types of sensory hairs have been described in the crayfish species, *Astacus leptodactylus*: smooth conical hairs and feathered hairs, which are evenly distributed along the flagellum. Both hairs are sensitive to low amplitude vibrations, with smooth hairs being stimulated directly by motion in the water, whereas feathered hairs are driven by the bending of the flagellum caused by the water movement [24]. These two types of hairs on the flagellum may aid in the localization of moving objects in the crayfish’s environment. This possibility is supported by findings that show crayfish in T-mazes with one denervated antenna turn towards their unaltered side, which suggests that bilateral comparisons of antenna signals are used to localize the source of water motion [65]. In addition to specialized appendages equipped with sensory hairs, crayfishes have mechanoreceptive hairs distributed over most of their bodies that respond to hydrodynamic disturbances [66]. In *Cherax destructor*, sensory hairs grouped together in pits found on the chelae are most sensitive to water vibration frequencies between 150 and 300 Hz [25]. Highly sensitive detectors can identify changes in the crayfish’s environment quickly, such as approaching predators from further distances. This can enable a faster behavioral response to change course or avoid potential predators, ultimately being a key sensory mechanism for survival.

***Terrestrial vertebrates (Rodentia and Carnivora).*** Mice (*Mus musculus*) and rats (*Rattus norvegicus*) have served as conventional animal models for studying the role of vibrissae in orienting and foraging under low light conditions [67]. Vibrissae located on the mystacial pad of the face are arranged in a grid-like pattern, consisting of rows and columns, where each individual whisker can be identified by a unique set of coordinates [6,19,68]. Rodent mystacial vibrissae are involved in both passive and active sensing [6,69,70]. Rodents interact with their environment when their whiskers contact an object or are displaced. Rodents also employ the use of their whiskers to detect and identify objects and surfaces of different shapes and textures by actively and rhythmically moving their whiskers [68]. This behavior is referred to as ‘whisking’ [6,71,72], and is used during a wide range of behaviors, including navigation and foraging, as noted above. Rats can use small (micro) and large (macro) vibrissae to explore and discriminate objects. Performing in texture discrimination tasks, rats temporally synchronize the movement of their microvibrissae placements with macrovibrissae whisking movements, providing continuous sensory input that can collectively probe the physical properties of the object [70]. Further, the movements of facial vibrissae can be consecutively organized, indicating the ability to further refine sensory information through movements over time. Seminal work in this field has revealed important insights to the mechanics of whisker transduction, such as the resonance properties of these vibrissae being key for detecting the boundaries of objects. Normal whisking tends to occur at frequencies between 5 and 15 Hz [73], but observed resonant frequencies are between 27 and 260 Hz depending on hair length, which may facilitate the transduction of mechanical stimuli during detection and orienting behaviors [74].

Further, the physical structure and stiffness of mechanosensory hairs can also contribute to sensitivity in the detection of stimuli. Young’s modulus (YM) measurements have revealed similar stiffness of mechanosensory hairs in rats, wandering spiders, and bats. In rat vibrissae, YM is between 3.34 ± 1.48 GPa across all segments of vibrissae, with the tip-segments being 3.96 ± 1.60 GPa and base-segments being 2.90 ± 1.25 GPa [75]. Trichobothria in the spider, *Cupiennius salei,* have a YM of 4 GPa [76], and big brown bats, *E. fuscus,* have an average YM of about 4.4 GPa [23].

The physical and mechanical properties of rodent vibrissae have been key in revealing potential technological applications of these biological sensors [2]. Rat vibrissae are some of the most well-studied sensory hair structures, and discoveries from detailed studies of these animal mechanosensors can serve to inspire further comparative work on the physical properties of hairs in both vertebrate and invertebrate species (see Section 5).

While whiskers provide tactile information, allowing rodents to successfully interact with objects and navigate in their environment, recent studies have demonstrated that vibrissae also signal displacement caused by airflow. Yu et al. [6] investigated the role of rat facial vibrissae in airflow sensing and characterized the mechanical responses to airflow. Individual whiskers were plucked and secured to an experimental setup to measure whisker movement. Two high-speed video cameras recorded movement of the whisker driven by naturalistic airflow stimuli. They found that whiskers bend in the direction of the airflow stimulus and that the bending magnitude is positively correlated with airflow speed [6]. In a behavioral study that further examined the role of airflow sensing in rat facial vibrissae, it was found that rats could localize airflow stimuli emitted from one of five fans in an arena [69]. Notably, rats significantly declined in their performance of the localization task following bilateral removal of facial vibrissae. More recent work has further investigated vibrissal airflow sensing and found that the direction and magnitude of the whisker’s deflection changes as a function of airflow speed [77]. In addition, they performed recordings in primary sensory trigeminal ganglion neurons to vibrissal stimulation and report that the firing rate of these neurons increased with airspeed, suggesting that rodent facial vibrissae can mediate tactile and anemotaxic behavior [6,69,77].

Other rodent species, such as hamsters, gerbils, chinchillas, and naked mole-rats, also possess sensory hairs that function as mechanosensors for tactile-guided orienting and foraging [7,78,79,80]. While a majority of rodent mechanosensory research has focused on cranial or facial vibrissae, some rodent species have postcranial vibrissae, such as the naked mole-rat (*Heterocephalus glaber*) [7,79]. Naked mole-rats are subterranean rodents with poor visual and auditory acuity [7]. To navigate elaborate underground burrows, forage for food, and care for their young, naked mole-rats must rely on mechanosensors to guide their behavior. Like other underground mammals, the naked mole-rat has a highly specialized somatosensory system designed to aid in navigation in low light conditions. In addition to an array of facial vibrissae, naked mole-rats are equipped with a unique array of approximately 40 postcranial vibrissae along the body [7,79]. These body vibrissae are sparsely and systematically distributed in a grid-like pattern from the torso, all the way to the tail. Previous work has shown that the body vibrissae play a role in tactile guided sensing. One study found that the deflection of a single body vibrissa of an unrestrained naked mole-rat elicited orienting behaviors [79]. Specifically, stimulation of the body vibrissa caused the animal to orient its snout in the direction of the stimulation, revealing that the body vibrissae enable the animal to accurately localize and orient to stimuli in the environment. As part of this study, two additional experiments were conducted to examine responses to stimulation of other tactile receptors in the skin and facial vibrissae. In the first experiment, the skin between body vibrissae was stimulated. They observed that skin stimulation was less reliable in eliciting orienting responses and did not always evoke the animal’s orientation towards the site of stimulation. In the second experiment, they found that when the facial vibrissae were deflected, the animal exhibited a snapping movement, which was not present during body vibrissae stimulation. Taken together, these findings demonstrate that body vibrissae of the naked mole-rat, much like facial vibrissae in other animals, serve as key mechanosensors and support the role of these sensory hairs in tactile guided detection and orientation.

Like rodents, cats (*Felis catus*) have been a key model species for studying the role of sensory hairs in exploration and navigation, particularly in low-light conditions [81,82,83]. The facial vibrissae of cats are large, tapered structures that activate a rich variety of mechanoreceptors upon deflection [83,84]. In a classic behavioral study, Schmidberger [81] compared blind cats navigating their environment with and without whiskers. It was found that cats whose whiskers had been removed tended to bump into objects in their surroundings more frequently than cats with intact whiskers. Further, their ability to locate small openings declined. When walking down a corridor, cats without whiskers walked at a slower speed and with impaired dexterity compared to cats with whiskers, suggesting whiskers played a key role in effective navigation, particularly in complex environments with obstacles. Interestingly, cats with intact whiskers not only successfully avoided obstacles, but they also were able to stop in time to avoid collisions when their whiskers came into contact with an object. In addition to facial vibrissae, cats possess carpal tactile hairs located on their forelimbs [85,86,87]. These hairs are located above the wrist on the volar side and are structurally similar and show properties resembling facial vibrissae. For both cats and rats, specialized hairs play a critical role in sensing and transmitting tactile information while moving through an environment.

***Aquatic vertebrates (Cetacea, Sirenia, Carnivora).*** Much like their terrestrial counterparts, aquatic mammals possess sensory hairs on their face and body. The distribution of hairs on the body and face varies across species, depending on their primary function. For example, Bowhead whales (*Balaena mysticetus*) have patches of hairs on their lips and caudal to their blowholes, which are thought to act as a passive sensory system to detect flow of water and air [26]. Another example is the Harbor seal (*Phoca vitulina*) that uses facial vibrissae to detect water movements created by prey [88,89].

Florida manatees (*Trichechus manatus latirostris*) are obligate aquatic mammals that inhabit warm, shallow waters with low visibility. Manatees have a poorly developed visual system and lack the ability to echolocate [90,91]. To successfully navigate their environments, they rely largely on sensory input from an array of facial hairs and bristles, as well as a system of postcranial hairs distributed over their bodies [10,29,92,93]. Through anatomical studies, these sensory hairs have been shown to share attributes with vibrissae found in other terrestrial species, which include prominent blood sinus complex, a capsule of dense connective tissue, and substantial innervation [29,92]. Research findings also suggest that the facial vibrissae of manatees are used in active touch, such as tactile exploration and feeding [94], whereas sensory hairs along the body of manatees have been hypothesized to play a role in passive detection of the environment via perturbations of the water [10,95]. 

Recent behavioral studies of manatees have further investigated the hypothesis that postcranial hairs are involved in detection of hydrodynamic stimuli. Gaspard et al. [10] conducted a series of experiments in which manatees were trained on a go/no-go task, where the goal was to correctly discriminate the directional flow of hydrodynamic stimuli. Manatees were trained to indicate the direction of a stimulus by withdrawing from a stationing bar and touching a response target with their muzzle on the side where a stimulus was presented. These experiments were conducted with postcranial hairs either intact or trimmed. Researchers observed that the manatee’s ability to detect and discriminate hydrodynamic stimuli was significantly attenuated after postcranial hairs were trimmed. These findings implicate this array of sensory hairs on the body of the manatee in the detection and localization of hydrodynamic stimuli, suggesting that these hairs can aid in exploration and navigation. 

***Shared functional properties: Navigation and exploration.*** Specialized sensory hairs have evolved to suit environmental constraints, such as low-light conditions, narrow or cluttered spaces, and turbulent winds or waters. Aquatic organisms often have shorter hairs with larger diameters to better suit the kinetics of water compared to hairs primarily exposed to air, such as those found in rodents. For instance, in manatees, the postcranial hairs are shorter and wider compared to the facial vibrissae (Table 1). Thus, hair morphology does not just evolve relative to functional needs behaviorally, but also relative to the physics of the environment they are in. All of the organisms discussed in this section possess highly sensitive hairs or hair-like structures that enable them to explore and navigate novel or complex environments, while avoiding potential threats. Crayfish are equipped with two different types of hairs along the flagellum that provide mechanosensory signals to guide behaviors, such as orienting and detection of fluid motion. Similarly, the naked mole-rat and the manatee possess both facial and postcranial vibrissae that enables them to accurately detect their surroundings. Both of these organisms rely on these hairs to orient in low visibility conditions on land and in water, respectively. Despite differences in the structure and location of hairs in species as diverse as crustacea, rodents, and manatees, the function of mechanosensory hairs appears largely conserved across organisms. 

Vibrissae on different body locations (i.e., face, body) and in different taxa serve as key mechanosensors for the detection and discrimination of objects in the environment, which are used by animals to navigate, forage, find mates, and evade predators. Knowledge of the rich array of biological sensors found throughout the animal kingdom can inform the design of artificial sensors that are used on robotic platforms tasked with the detection and discrimination of objects, while exploring novel environments.

## 4. Prey Capture and Feeding

Many organisms have evolved species-specific adaptations for searching, capturing, and consuming their prey. Hair and hair-like structures provide sensory feedback during foraging, as well as during the manipulation of food or prey.

***Terrestrial invertebrates (Araneae, Hymenoptera).*** With the vast diversity of invertebrates, specializations of hair and hair-like structures can provide key sensory and mechanical feedback that enables a wide variety of behaviors, including foraging, prey capture, and feeding. Many species of spiders that do not establish webs and instead roam to hunt their prey have particularly numerous hair sensilla that can support highly sensitive detection. For example, the nocturnal wandering spider, *Cupiennius salei,* waits for prey and then rapidly strikes to capture its target. Using sensory cues from substrate vibrations caused by creatures walking on the ground or air movements like those produced by flight, *C. salei* can detect their prey and then rapidly strike within a few hundred milliseconds ([15], overviewed in [96]). In addition to specialized sensory organs like the lyriform slit organ that are sensitive to vibrations within the environment [97,98], *C. salei* have specialized hair-like structures called trichobothria (filiform hairs, similar to those described in the section *Flying invertebrates*), support prey capture behavior in *C. salei*. The hairs are approximately 0.1 to 1.4 mm in length, with frequency responses ranging between about 40 Hz and 600 Hz, exhibiting among the highest sensitivities of a biological sensor currently known [14,15,16]. Interneurons receive sensory input from trichobothria on the walking legs of *C. salei*, with individual interneurons showing different response characteristics, suggesting parallel processing of different parameters of the sensory signal across populations of neurons [99]. The phasic response characteristics of the receptor cells of the trichobothria and interneurons are particularly suited for detecting behaviorally relevant pulse-like air flow, such as those caused by small prey flying [99,100,101]. Specifically, pulse-like airflow can be distinguished from background noise and low velocity airflow with relatively small fluctuations [99,100], making the sensitivities and response patterns of trichobothria specialized for detecting and locating prey. *C. salei* are covered in these specialized sensory structures, creating a sensory array that can detect tiny changes in fluid flow [14,102]. The powerful sensitivity of these hairs as well as their physical positioning on the body of these spiders makes them a powerful predator able to detect and capture small flying prey rapidly and accurately. 

**Figure 3:**
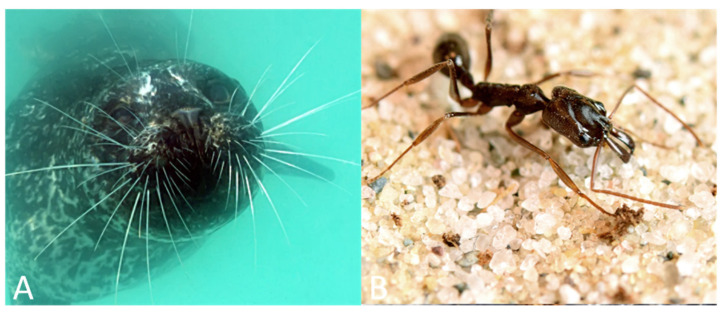
Hairs and hair-like structures aid in prey capture and foraging. (**A**) Harbor seal (*Phoca vitulina*) facial vibrissae, with a caudal curvature to their whiskers. These hairs aid in both navigation and foraging to detect the movement of prey (image modified from [109]). (**B**) Worker trap jaw ants (*Odontomachus brunneus*) use their powerful mandibles to capture food or escape danger. Hair triggers enable rapid closure of these mandibles image modified from [110]).

The sensitivity and rapid sensory feedback carried by specialized sensory hairs supports diverse feeding behaviors in many terrestrial insects. The trap jaw ant (genus *Odontomachus*), for instance, has mandibles that can strike in less than 0.5 ms, with the entire reflex from sensory stimulation to strike taking between 3 and 10 ms [103,104,105,106]. In one species of trap jaw ants, *Odontomachus bauri,* predatory strikes close at speeds between 35 and 64 m/s, making it one of the fastest ballistic predatory appendages in the animal kingdom [107]. Two very long bristles (600–1200 µm) located on each mandible act as mechanosensory triggers that release the trap jaw mechanism, leading to the rapid mandible strike [17]. These long bristles have large afferent axons that rapidly provide sensory feedback indicating an object is within striking range and coordinate a synchronized closure of the mandibles [104]. Interestingly, this hair trigger requires sufficient behavioral context to snap the mandibles closed. The mandible strike response is inhibited in the presence of conspecifics [17,108], indicating this behavior is not simply triggered by the stimulation of these hairs alone and instead requires proper sensory and behavioral conditions to elicit this powerful strike (see Figure 3).

In addition to predation, the trap jaw ant’s remarkable mandibles can also be used for propulsion. These ants can orient their mandibles against substrates to launch themselves into the air, a mechanism that improves the likelihood of survival when escaping from predators [110,111]. Escape jumps can reach vertical heights of 6–8 cm, and defensive jumps reach horizontal distances of 5–40 cm [107]. Various sizes of hairs and hair-like structures provide necessary sensory information to coordinate the rapid movements of these mandibles. Specifically, in addition to the long bristles (i.e., trigger hairs), the mandibles also possess very small hair-like sensilla and a row of smaller hairs that likely provide proprioceptive information about the positioning of the mandibles [17].

***Aquatic vertebrates (Carnivora, Sirenia).*** The sensory hairs or vibrissae of marine mammals serve as mechanosensors and show species-specific specializations for foraging. These specializations depend on a variety of factors, including the animal’s environment, diet, and morphology. For example, the northern elephant seal (*Mirounga angustirostris*) forages in deep waters, both during the day and at night, and feeds primarily on vertically migrating prey, such as plankton, fish, and squid [11]. While the northern elephant seal has high visual sensitivity, it is limited by the time of day in which it forages. In low light conditions, the northern elephant seal must rely on multimodal sensing and use both vision and mechanosensation via its facial vibrissae to forage for prey [11]. The Harbor seal (*Phoca vitulina*) uses its facial vibrissae to detect changes in waterflow to navigate (see Section 3) and also to track the movement of prey [27], which is aided by the structure of this species’ vibrissae that reduces self-generated noise [109] (see Figure 3).

Another aquatic mammal that utilizes sensory hairs to forage and feed is the Pacific walrus (*Odobenus rosmarus divergens*) [10,112,113]. The Pacific walrus forages at night in deep water with low visibility and preys on benthic organisms, such as clams, oysters, and mussels [112,113]. Due to the position of its eyes and the width of its snout, the Pacific walrus has reduced visibility in front of its face [113] and therefore takes advantage of sensory hairs located on the face for tactile information from the surroundings. The Pacific walrus has approximately 400 to 700 vibrissae organized into 13 to 18 rows on their mystacial pads [110]. These vibrissae are extremely mobile and have been observed to be active and move rapidly during the exploration of objects or during feeding [112]. Early research hypothesized that these vibrissae in Pacific walruses serve a sensorimotor function and were responsible for providing crucial tactual information for foraging and feeding. One study demonstrated that even when blindfolded, a walrus could discriminate objects of different shapes and sizes using the mystacial vibrissae [113]. Additionally, they found that vibrissae on different parts of the mystacium served different roles, where the lateral vibrissae functioned primarily for detection and the more central vibrissae for discrimination. 

In addition to tactile sensing, vibrissae have also been shown to be involved in the handling of objects and food. Sirenians are the only mammals known to use mystacial vibrissae for tactile exploration and object manipulation. As noted above, the Florida manatee utilizes facial vibrissae, also referred to as perioral bristles, for tactile exploration and feeding, as well as oripulation, the handling of objects and food with facial musculature [92,94,114]. This behavior was first described in 1875 by Chapman [115] and has since been studied to define the range of control of the facial vibrissae. Marshall et al. [94] studied how Florida manatees used their perioral bristles to interact with and oripulate objects and food. In this study, manatees were given a variety of vegetation and inanimate objects during feeding trials. The researchers observed that manatees primarily relied on tactile information from their bristles to guide feeding behaviors. Specifically, they reported that manatees tended to close their eyes while foraging and feeding and thought it may be a protective measure to avoid damaging their eyes from vegetation. Moreover, they found that the use of the bristles varied depending on whether vegetation was submerged or floating and manatees could independently reverse the direction of specific bristles when presented with a food item or object that they disliked. The vibrissal-muscular complex enables coordinated and rhythmic movements of the lips, bristles, and jaw, allowing for dexterous exploration and manipulation of objects in the environment. These findings support the role of perioral bristles in both tactile discrimination and prehensile control for foraging and feeding.

***Shared functional properties: Prey capture and feeding.*** In some of the examples we have discussed in this section, mechanosensory hairs function to enable highly specialized foraging and feeding strategies. Speedy responses from these hairs provides the necessary sensory input to enable some of the fastest biological movements observed in the animal kingdom. The trichobothria of *Cupiennius salei* is an interesting biological model for detecting behaviorally relevant sensory inputs because they show the highest sensitivities of any biological sensor currently known [14,15,16]. Trap-jaw ants like *Odontomachus bauri* rely on microscopic trigger hairs to execute one of the fastest ballistic predatory motions in the animal kingdom. Both examples of invertebrates presented here show rapid responses, with *O. bauri* receiving sensory input from specialized trigger hairs that directly innervates muscles in the jaw and the trichobothria of *C. salei* detecting pulse-like air flow produce by small flying prey. Compared to the highly sensitive hairs and hair-like structures of invertebrates, sensory hairs of vertebrates tend to be much larger (Table 1) while still providing mechanosensory signals used for foraging and feeding. In aquatic mammals, such as walruses and manatees, facial vibrissae or bristles provide sensory signals to detect and discriminate prey from their surroundings, as well as guiding manipulation of food items. Manatee mechanosensing also operates with a balance of sensitivity and accuracy to find and handle prey.

The examples of biological sensors illustrate species-specific adaptations that enable natural foraging and feeding behaviors across the animal kingdom. A deeper understanding of animal mechanosensors in foraging can inform technological advances in a variety of applications, such as collecting samples from the ocean floor.

## 5. Engineering Applications of Biologically Inspired Hair Sensors

Mechanosensors are essential for the survival of all living animals, including humans. Many technological advances have been influenced by scientific knowledge of biological mechanosensors throughout the animal kingdom, from invertebrates to mammals, including the development and implementation of biomimetic hair and hair-like sensors. As discussed in this review, animals utilize sensory hairs to locomote, navigate, forage, and interact with their environment. Many bio-inspired robots have been developed based on the specialized functional properties of sensory hairs in animals. In this section, we present some examples in which natural hair and hair-like sensors have inspired technology thus far and propose new applications.

***Selected examples of bioinspired hairs in technology applications*.** In 2007, Pearson et al. [116] designed the Whiskerbot, a biologically inspired robot that implemented a rodent whisker sensory system for exploration. The Whiskerbot is made of a “head” sensory unit with two rows of three whiskers on each side, and a two-wheeled “body”. To mimic the whisking behavior of rodents, each whisker shaft can sweep forward and backward, and the angle of each shaft can be measured with respect to the head unit using optical shaft encoders. The Whiskerbot is equipped with three types of functions: dead reckoning, exploring the environment, and orienting to the stimulus. While the implementation of dead reckoning is based on conventional path integration and exploring the environment is hard-wired into the robot to mimic the exploratory strategy of rodents, exploring and orienting to a stimulus is achieved by contacts made on the whisker shaft. Other functions of the rodent whisking system have been implemented in sensory robotics [2,3,116,117,118], namely the extraction of spatial and textural features of the environment, even in small, low-light conditions [71].

Biomimetic sensors inspired by other characteristics and functional properties of rodent whiskers have been developed. In 2014, Takei et al. [117] developed electronic whiskers, also referred to as e-whiskers. These whiskers were designed to detect changes in pressure and strain and were constructed from carbon nanotube (CNT) to provide flexibility and silver nanoparticles (AgNPs) to enhance conductivity. Testing revealed that an array of e-whisker sensors successfully mapped air flow in two and three dimensions [117,118].

Hair-like sensors found in arthropods have also served as inspiration for technology, because of their high sensitivity, small size, and role in detecting changes in fluid (i.e., air and water) dynamics. For example, Ko et al. [119] designed an acceleration sensor inspired by insect filiform hairs. They attached a rigid metal rod to a piezoresistive membrane that detects changes in electrical resistance when applied with physical force, acting as a strain sensor. Numerous groups have also developed highly sensitive artificial hair-like sensors capable of detecting changes in flow velocity, direction, and strength [4,119,120].

***Inspiration of animal mechanical hairs in future technology applications.*** Artificial sensors in robotics can continue to improve in sensitivity, dexterity, efficiency, and accuracy, drawing from knowledge of biological sensory hairs and hair-like structures. Below, we propose a few curated examples of instances where technology and biology can intersect in mutually beneficial efforts.

Developed in 1975, robotic grippers are tasked with securely grasping objects, a common operation for robotic manipulators. A gripper can be defined as a tool that is mounted at the end of a piece of equipment to grasp, carry, and place objects. While grasping objects may appear to be executed with ease by many animals, including humans, this operation has proven difficult for robots. In addition to grasping an object, robotic grippers must also have the ability to sense the characteristics of the object (i.e., shape, size, material) and interact with the environment in order to adapt their grasp to prevent crushing an object and to avoid dropping it [121,122,123]. Various sensors, including tactile, visual, and hearing sensors, have been integrated into robotic grippers to enhance sensitivity and stability. We propose that robotic grippers could benefit from an artificial hair-like mechanosensor to enhance performance. Specifically, the application of hair-like sensors to robotic grippers could allow for earlier detection of an object’s position, as well as changes to the surrounding environment (i.e., fluid dynamics and vibrations). Moreover, hair-like sensors could also contribute as an additional layer for monitoring the strength and effectiveness of the grasp.

As highlighted in this review, there are many animal behaviors that rely heavily on sensory feedback from hairs on their face and body, and a wider range of biologically inspired technology could implement this knowledge in robotic systems. For example, Colorado et al. [124] designed a micro aerial vehicle with morphing wings inspired by bat anatomy and flight. The robot was constructed from shape memory alloys, or SMAs, which act as muscle-like actuators, providing the motions of a bat’s wingbeat, as well as mimicking the flexible nature of the bat’s bone structure. The goal of this work was to develop the first autonomously flying bat-like robot. While many features of bat anatomy and physiology were applied in the development of this flying robot, Colorado et al. [124] omitted a key sensor, airflow sensing hairs on the wings. The implementation of artificial hair-like sensors on their flying bat-like robot could enhance aerodynamic performance.

Another example of robotic systems that could benefit from hair-like sensors are small-scale drones. Many advances have been made in the technology for small-scale drones that are used for a range of applications, from photography to environmental monitoring and mapping [125]. One limitation of small-scale drones is poor flight control in turbulent conditions [125]. Wind gusts and turbulence can lead to stall, which both drones and flying animals alike encounter. As discussed above, bats have specialized wing hair sensors to detect changes in airflow across their wings [58]. The incorporation of bio-inspired hair-like sensors could allow for increased sensitivity in airflow detection, and in turn result in enhanced flight control in small-scale drones.

Research that has provided insights into the mechanisms behind sensory properties of hairs and hair-like structures have been crucial to the improvement of bioinspired technologies. For example, work on rat whisking revealed the importance of active vibrissa movement control and sensory encoding of vibrissa deflection, which inspired artificial whisker sensors in land-based robots and autonomous underwater vehicles [2]. Further modeling, simulation, and behavioral techniques have revealed key insights to the contribution of vibrissa movement properties and shape to sensory information transduction [126,127]. The rigorous work on rat vibrissae exemplifies the potential impact of biological research on artificial sensor development and application. Many yet understudied hair and hair-like sensors in the animal kingdom hold the potential for novel applications in robotics and other technologies. Further biological research focused on the physical characteristics of natural sensory hairs and their roles in animal behaviors can inform advances in the development and optimization of artificial hair-like sensors.

***Current technology challenges and limitations.*** While there have been significant strides in the advancement of bioinspired hair sensor technology, the field faces many challenges and limitations. One key limitation arises from the current materials and fabrication techniques used to construct artificial mechanosensors. Natural hairs and hair-like structures are often small, even microscopic in some animals, making them difficult to mimic. In addition to their size, biological hair and hair-like structures are highly sensitive, and composed of flexible, and strong materials, such as cuticle in invertebrates [12,15,24] and keratin in mammals [128]. Implementation of all these properties in a single sensor or array of sensors poses a challenge for engineers. Depending on the material, it may not be feasible to construct a sensor that has the exact same size and sensitivity as the biological one. For instance, there is often a hysteresis effect, or lag, when using polymers to design hair-like sensors [4].

Another limitation of artificial hair-like sensors is their durability. Animals encounter dramatic changes in their environmental conditions, such as extreme winds and fluctuations in temperature and weather conditions, and the sensory hairs and hair-like structures they possess must also withstand these changes. In the case of flying invertebrates, such as locusts, and flying vertebrates, such as bats, mechanosensory hairs that are crucial for airflow sensing and flight control must endure turbulent winds. Comparably, aquatic animals that possess specialized sensory hairs may also experience drastic changes in water conditions, such as temperature and currents. Designing an artificial sensor that is both sensitive and durable under a wide range of conditions has proven difficult, with the biological mechanosensors outperforming the artificial sensors, and many of the artificial sensors becoming damaged during testing. Novel materials may aid in overcoming limitations in artificial sensors.

## 6. Conclusions

While many inherent specializations of biological systems have yet to be uncovered, new discoveries of biological sensors continue to motivate and innovate new technologies. Our review aims to highlight the diversity of sensory hair and hair-like structures in the animal kingdom and their functions in supporting a rich repertoire of behaviors, which can inform and inspire advances in sensing technology. By better understanding the properties of natural mechanosensory hairs, such as their morphology, role in behaviors, and the feedback they provide to actuators, we can develop sensors with enhanced sensitivity, durability, and functionality. The biological and artificial systems can also reciprocally advance science and engineering, whereby detailed understanding of biological systems can inform technology, and artificial systems can reveal gaps in knowledge of biological systems. Together, these two fields can synergistically inform future advances in sensory-guided actions. 

## Figures and Tables

**Table 1:** Overview of features of hairs and hair-like structures.

Category	Order	Species	Hair Structure	Hair Length (mm)	Location	Function
Terrestrial Invertebrates	Diptera	Brachycera (fly)	Setae	0.0339 ± 0.00471 [12]	Basal part of pulvillus	Attachment to surfaces
	Blattodea	*Periplaneta americana* (Cockroach)	Sensilla of hair plates (short/long)	Short: 0.005–0.03 Long: 0.03–0.07 [13]	Leg joints	Limit detectors for coordinated movements
	Aranneae	*Cupiennius salei* (Wandering spider)	Trichobothria (filiform hairs)	0.1–1.4 [14,15,16]	Body/legs	Prey detection
	Hymenoptera	*Odontomachus bauri* (trap jaw ant)	Hair-like sensilla/bristles	0.6–1.2 [17]	Mandibles	Prey detection
Terrestrial Vertebrates	Carnivora	*Felis catus* (Domestic cat)	Capral vibrissae	10–20 [18]	Forelimbs	Coordinated movement
	Carnivora	*Felis catus* (Domestic cat)	Mystacial vibrissae	40–70 [18]	Face	Coordinated movement
	Rodentia	*Mus musculus* (Mouse)	Mystacial vibrissae	30 [19]	Face	Coordinated movement
	Rodentia	*Rattus norvegicus domestica* (rat)	Mystacial vibrissae	10–60 [6]	Face	Coordinated movement
Flying Invertebrates	Orthoptera	Locusts	Trichoid sensilla	0.03–0.35 [20]	Head capsule	Airflow sensing
	Orthoptera	Crickets	Cerci	0.03–1.5 [21,22]	Body	Airflow sensing
Flying Vertebrates	Chiroptera	*Eptesicus fuscus* (Big brown bat)	Sensory hairs	0.08–1 [23]	Wing/tail membrane	Airflow sensing
Aquatic Invertebrates	Decapoda	*Astacus leptodactylus* (Crayfish)	Conical/ Feathered hairs	Conical: 0.4–0.8 Feathered: 0.9–1.2 [24]	Flagellum of antennae	Fluid sensing
	Decapoda	*Cherax destructor* (Crayfish)	Sensory hairs	0.02 [25]	Chelae	Fluid sensing
Aquatic Vertebrates	Cetacea	*Balaena mysticetus* (Bowhead whale)	Sensory hairs	3–46 [26]	Lips, caudal blowhole	Fluid sensing
	Carnivora	*Mirounga angustirostris* (Northern elephant seal)	Facial vibrissae	7.54–138.14 [11]	Face	Foraging
	Carnivora	*Phoca vitulina* (Harbor seal)	Facial vibrissae	22.9 [27]	Face	Fluid sensing, foraging
	Sirenia	*Trichechus manatus latirostris* (Florida manatee)	Facial vibrissae/bristles	1–10 [28]	Face	Foraging, detection, discrimination,
	Sirenia	*Trichechus manatus latirostris* (Florida manatee)	Postcranial vibrissae	2–9 [29]	Body	Detection and localization

## Data Availability

Not applicable.

## References

[1] Pfeifer R., Lungarella M., Iida F. (2007). Self-Organization, Embodiment, and Biologically Inspired Robotics. Science.

[2] Solomon J.H., Hartmann M.J. (2006). Robotic whiskers used to sense features. Nature.

[3] Solomon J., Hartmann M. (2008). Artificial Whiskers Suitable for Array Implementation: Accounting for Lateral Slip and Surface Friction. IEEE Trans. Robot..

[4] Han Z., Liu L., Wang K., Song H., Chen D., Wang Z., Niu S., Zhang J., Ren L. (2018). Artificial Hair-Like Sensors Inspired from Nature: A Review. J. Bionic Eng..

[5] Asterindi Blandin A.A., Bernardeschi I., Beccai L. (2018). Biomechanics in Soft Mechanical Sensing: From Natural Case Studies to the Artificial World. Biomimetics.

[6] Yu S.W.Y., Graff M.M., Hartmann M.J.Z. (2016). Mechanical responses of rat vibrissae to airflow. J. Exp. Biol..

[7] Crish C.M., Crish S.D., Comer C., Prescott T., Ahissar E., Izhikevich E. (2016). Tactile Sensing in the Naked Mole Rat. Scholarpedia of Touch.

[8] Weis-Fogh T. (1949). An Aerodynamic Sense Organ Stimulating and Regulating Flight in Locusts. Nature.

[9] Sterbing-D’Angelo S.J., Chadha M., Marshall K.L., Moss C.F. (2017). Functional role of airflow-sensing hairs on the bat wing. J. Neurophysiol..

[10] Gaspard J.C., Bauer G.B., Mann D.A., Boerner K., Denum L., Frances C., Reep R.L. (2017). Detection of hydrodynamic stimuli by the postcranial body of Florida manatees (Trichechus manatus latirostris). J. Comp. Physiol. A.

[11] Mcgovern K.A., Marshall C.D., Davis R.W. (2015). Are Vibrissae Viable Sensory Structures for Prey Capture in Northern Elephant Seals, Mirounga angustirostris?. Anat. Rec. Adv. Integr. Anat. Evol. Biol..

[12] Gorb S.N. (1998). The design of the fly adhesive pad: Distal tenent setae are adapted to the delivery of an adhesive secretion. Proc. R. Soc. B Boil. Sci..

[13] Wong R.K., Pearson K.G. (1976). Properties of the trochanteral hair plate and its function in the control of walking in the cockroach. J. Exp. Biol..

[14] Barth F.G. (2014). The slightest whiff of air: Airflow sensing in arthropods. Flow Sensing in Air and Water.

[15] Barth F.G. (2002). Spider senses–technical perfection and biology. Zoology.

[16] Barth F.G. (2004). Spider mechanoreceptors. Curr. Opin. Neurobiol..

[17] Gronenberg W., Tautz J. (1994). The sensory basis for the trap-jaw mechanism in the ant Odontomachus bauri. J. Comp. Physiol. Ser. A.

[18] Gottschaldt K.-M., Iggo A., Young D.W. (1973). Functional characteristics of mechanoreceptors in sinus hair follicles of the cat. J. Physiol..

[19] Brecht M., Preilowski B., Merzenich M.M. (1997). Functional architecture of the mystacial vibrissae. Behav. Brain Res..

[20] Taylor G.K., Krapp H.G. (2007). Sensory Systems and Flight Stability: What do Insects Measure and Why?. Advances in Insect Physiology.

[21] Chiba A., Kämper G., Murphey R.K. (1992). Response Properties of Interneurons of the Cricket Cercal Sensory System are Conserved in Spite of Changes in Peripheral Receptors during Maturation. J. Exp. Biol..

[22] Jacobs G.A., Miller J.P., Aldworth Z. (2008). Computational mechanisms of mechanosensory processing in the cricket. J. Exp. Biol..

[23] Sterbing-D’Angelo S.J., Liu H., Yu M., Moss C.F. (2016). Morphology and deflection properties of bat wing sensory hairs: Scanning electron microscopy, laser scanning vibrometry and mechanics model. Bioinspir. Biomim..

[24] Tautz J., Masters W.M., Aicher B., Märkl H. (1981). A new type of water vibration receptor on the crayfish antenna. J. Comp. Physiol. A.

[25] Tautz J., Sandeman D.C. (1980). The Detection of Waterborne Vibration by Sensory Hairs on the Chelae of the Crayfish. J. Exp. Biol..

[26] Drake S.E., Crish S.D., George J.C., Stimmelmayr R., Thewissen J. (2015). Sensory Hairs in the Bowhead Whale, Balaena mysticetus (Cetacea, Mammalia): Sensory Hairs in the Bowhead Whale. Anat. Rec. Adv. Integr. Anat. Evol. Biol..

[27] Dougill G., Starostin E.L., Milne A.O., Van Der Heijden G.H.M., Goss V.G.A., Grant R.A. (2020). Ecomorphology reveals Euler spiral of mammalian whiskers. J. Morphol..

[28] Reep R.L., Marshall C.D., Stoll M.L., Whitaker D.M. (1998). Distribution and Innervation of Facial Bristles and Hairs in the Florida Manatee (Trichechus Manatus Latirostris). Mar. Mammal. Sci..

[29] Reep R.L., Marshall C.D., Stoll M.L. (2002). Tactile Hairs on the Postcranial Body in Florida Manatees: A Mammalian Lateral Line?. Brain Behav. Evol..

[30] Miller J.P., Krueger S., Heys J.J., Gedeon T. (2011). Quantitative Characterization of the Filiform Mechanosensory Hair Array on the Cricket Cercus. PLoS ONE.

[31] Pringle J.W.S. (1937). Proprioception in Insects I: A New Type of Mechanical Receptor from the Palps of the Cockroach. J. Exp. Biol..

[32] Tuthill J.C., Wilson R.I. (2016). Mechanosensation and Adaptive Motor Control in Insects. Curr. Biol..

[33] Dean J., Wendler G. (1983). Stick Insect Locomotion on a Walking Wheel: Interleg Coordination of Leg Position. J. Exp. Biol..

[34] Kuenzi F., Burrows M. (1995). Central connections of sensory neurones from a hair plate proprioceptor in the thoraco-coxal joint of the locust. J. Exp. Biol..

[35] Büschges A. (2005). Sensory Control and Organization of Neural Networks Mediating Coordination of Multisegmental Organs for Locomotion. J. Neurophysiol..

[36] Borgmann A., Hooper S.L., Büschges A. (2009). Sensory Feedback Induced by Front-Leg Stepping Entrains the Activity of Central Pattern Generators in Caudal Segments of the Stick Insect Walking System. J. Neurosci..

[37] Knebel D., Wörner J., Rillich J., Nadler L., Ayali A., Couzin-Fuchs E. (2018). The subesophageal ganglion modulates locust inter-leg sensory-motor interactions via contralateral pathways. J. Insect Physiol..

[38] Knebel D., Rillich J., Nadler L., Pflüger H.-J., Ayali A. (2019). The functional connectivity between the locust leg pattern generators and the subesophageal ganglion higher motor center. Neurosci. Lett..

[39] Knebel D., Ayali A., Pflüger H.-J., Rillich J. (2017). Rigidity and Flexibility: The Central Basis of Inter-Leg Coordination in the Locust. Front. Neural Circuits.

[40] Gorb S.N., Beutel R.G., Gorb E.V., Jiao Y., Kastner V., Niederegger S., Popov V.L., Scherge M., Schwarz U., Vötsch W. (2002). Structural Design and Biomechanics of Friction-Based Releasable Attachment Devices in Insects. Integr. Comp. Biol..

[41] Bullock J.M.R., Drechsler P., Federle W. (2008). Comparison of smooth and hairy attachment pads in insects: Friction, adhesion and mechanisms for direction-dependence. J. Exp. Biol..

[42] Van Griethuijsen L.I., van Trimmer B.A. (2014). Locomotion in caterpillars. Biol. Rev..

[43] Weeks J.C., Jacobs G.A. (1987). A reflex behavior mediated by monosynaptic connections between hair afferents and motoneurons in the larval tobacco hornworm, Manduca sexta. J. Comp. Physiol. A.

[44] Newland P. (1991). Physiological properties of afferents from tactile hairs on the hindlegs of the locust. J. Exp. Biol..

[45] Shimozawa T., Murakami J., Kumagai T., Barth F.G., Humphrey J.A.C., Secomb T.W. (2003). Cricket Wind Receptors: Thermal Noise for the Highest Sensitivity Known. Sensors and Sensing in Biology and Engineering.

[46] Boyd K., Ewer D.W. (1949). Flight Responses in Grasshoppers. S. Afr. Sci..

[47] Weis-Fogh T., Pringle J.W.S. (1956). Biology and physics of locust flight IV. Notes on sensory mechanisms in locust flight. Philos. Trans. R. Soc. London Ser. B Biol. Sci..

[48] Camhi J.M. (1970). Yaw-Correcting Postural Changes in Locusts. J. Exp. Biol..

[49] Camhi J.M. (1970). Sensory Control of Abdomen Posture in Flying Locusts. J. Exp. Biol..

[50] Neese V. (1965). Zur Funktion der Augenborsten bei der Honigbiene. J. Comp. Physiol. A.

[51] Boyan G.S., Ashman S., Ball E.E. (1986). Initiation and modulation of flight by a single giant interneuron in the cercal system of the locust. Naturwissenschaften.

[52] Landolfa M., Jacobs G. (1995). Direction sensitivity of the filiform hair population of the cricket cereal system. J. Comp. Physiol. A.

[53] Gnatzy W., Tautz J. (1980). Ultrastructure and mechanical properties of an insect mechanoreceptor: Stimulus-transmitting structures and sensory apparatus of the cereal filiform hairs of Gryllus. Cell Tissue Res..

[54] Fraser P.J. (1977). Cercal ablation modifies tethered flight behaviour of cockroach. Nature.

[55] Leem J.W., Willis W.D., Chung J.M. (1993). Cutaneous sensory receptors in the rat foot. J. Neurophysiol..

[56] Manfredi L.R., Baker A.T., Elias D.O., Dammann J.F., Zielinski M., Polashock V.S., Bensmaia S.J. (2012). The Effect of Surface Wave Propagation on Neural Responses to Vibration in Primate Glabrous Skin. PLoS ONE.

[57] Johansson R.S., Vallbo B. (1983). Tactile sensory coding in the glabrous skin of the human hand. Trends Neurosci..

[58] Sterbing-D’Angelo S., Chadha M., Chiu C., Falk B., Xian W., Barcelo J., Zook J.M., Moss C.F. (2011). Bat wing sensors support flight control. Proc. Natl. Acad. Sci. USA.

[59] Kang C., Reep R. (2013). Post-Cranial Hairs in Four Families of Bats. Acta Chiropterologica.

[60] Maxim H. (1912). Preventing Collisions at Sea: A Mechanical Application of the Bat’s Sixth Sense. Sci. Am..

[61] Zook J., Fowler B. (1986). A Specialized Mechanosensory Array of the Bat Wing. Myotis.

[62] Marshall K.L., Chadha M., Desouza L.A., Sterbing-D’Angelo S.J., Moss C.F., Lumpkin E.A. (2015). Somatosensory Substrates of Flight Control in Bats. Cell Rep..

[63] Casas J., Liu C., Krijnen G., Bhushan B. (2012). Biomimetic Flow Sensors. Encyclopedia of Nanotechnology.

[64] Sandeman D.C. (1985). Crayfish antennae as tactile organs: Their mobility and the responses of their proprioceptors to displacement. J. Comp. Physiol. A.

[65] McMahon A., Patullo B.W., Macmillan D.L. (2005). Exploration in a T-Maze by the CrayfishCherax destructorSuggests Bilateral Comparison of Antennal Tactile Information. Biol. Bull..

[66] Tazaki K., Ohnishi M. (1974). Responses from Tactile Receptors in the Antenna of the Spiny Lobster *Panulirus japonicus*. Comp. Biochem. Physiol. Part A Physiol..

[67] Vincent S.B. (1912). The Function of Vibrissae in the Behavior of the White Rat.

[68] Diamond M.E., von Heimendahl M., Knutsen P.M., Kleinfeld D., Ahissar E. (2008). Where and what in the whisker sensorimotor system. Nat. Rev. Neurosci..

[69] Yu Y.S.W., Graff M.M., Bresee C.S., Man Y.B., Hartmann M.J.Z. (2016). Whiskers aid anemotaxis in rats. Sci. Adv..

[70] Hartmann M.J. (2001). Active Sensing Capabilities of the Rat Whisker System. Auton. Robot..

[71] Welker W. (1964). Analysis of Sniffing of the Albino Rat 1. Behaviour.

[72] Carvell G., Simons D. (1990). Biometric analyses of vibrissal tactile discrimination in the rat. J. Neurosci..

[73] Berg R.W., Kleinfeld D. (2003). Rhythmic Whisking by Rat: Retraction as Well as Protraction of the Vibrissae Is Under Active Muscular Control. J. Neurophysiol..

[74] Hartmann M.J., Johnson N.J., Towal R.B., Assad C. (2003). Mechanical Characteristics of Rat Vibrissae: Resonant Frequencies and Damping in Isolated Whiskers and in the Awake Behaving Animal. J. Neurosci..

[75] Quist B.W., Faruqi R.A., Hartmann M.J. (2011). Variation in Young’s modulus along the length of a rat vibrissa. J. Biomech..

[76] DeChant H.-E., Rammerstorfer F., Barth F. (2001). Arthropod touch reception: Stimulus transformation and finite element model of spider tactile hairs. J. Comp. Physiol. A Neuroethol. Sens. Neural Behav. Physiol..

[77] Yu Y.S.W., Bush N.E., Hartmann M.J.Z. (2019). Whisker Vibrations and the Activity of Trigeminal Primary Afferents in Response to Airflow. J. Neurosci..

[78] Wineski L.E. (1983). Movements of the cranial vibrissae in the Golden hamster (Mesocricetus auratus). J. Zool..

[79] Crish S.D., Rice F.L., Park T.J., Comer C.M. (2003). Somatosensory organization and behavior in naked mole-rats I: Vibrissa-like body hairs comprise a sensory array that mediates orientation to tactile stimuli. Brain Behav. Evol..

[80] Mitchinson B., Grant R.A., Arkley K., Rankov V., Perkon I., Prescott T.J. (2011). Active Vibrissal Sensing in Rodents and Mar-supials. Philos. Trans. R Soc. B Biol. Sci..

[81] Schmidberger G. (1932). About the Importance of Whiskers in Cats. Z. Vgl. Physiol..

[82] Nilsson B.Y. (1972). Effects of Sympathetic Stimulation on Mechanoreceptors of Cat Vibrissae. Acta Physiol. Scand..

[83] Schultz W., Galbraith G.C., Gottschaldt K.-M., Creutzfeldt O.D. (1976). A comparison of primary afferent and cortical neurone activity coding sinus hair movements in the cat. Exp. Brain Res..

[84] Williams C.M., Kramer E.M. (2010). The Advantages of a Tapered Whisker. PLoS ONE.

[85] Nilsson B.Y., Skoglund C.R. (1965). The Tactile Hairs on the Cat’s Foreleg. Acta Physiol. Scand..

[86] Nilsson B.Y. (1969). Structure and Function of the Tactile Hair Receptors on the Cat’s Foreleg. Acta Physiol. Scand..

[87] Fitzgerald O. (1940). Discharges from the sensory organs of the cat’s vibrissae and the modification in their activity by ions. J. Physiol..

[88] Dehnhardt G., Mauck B., Bleckmann H. (1998). Seal whiskers detect water movements. Nature.

[89] Dehnhardt G., Mauck B., Hanke W., Bleckmann H. (2001). Hydrodynamic Trail-Following in Harbor Seals (Phoca vitulina). Science.

[90] Mass A.M., Ketten D.R., Odell D.K., Supin A.Y. (2012). Ganglion Cell Distribution and Retinal Resolution in the Florida Manatee, Trichechus Manatus Latirostris. Anat. Rec. Adv. Integr. Anat. Evol. Biol..

[91] Bauer G.B., Colbert-Luke D., Gaspard J., Littlefield B., Fellner W. (2003). Underwater Visual Acuity of Florida Manatees (Trich-echus manatus latirostris). Int. J. Comp. Psychol..

[92] Reep R.L., Stoll M.L., Marshall C.D., Homer B.L., Samuelson D.A. (2001). Microanatomy of facial vibrissae in the Florida manatee: The basis for specialized sensory function and oripulation. Brain Behav. Evol..

[93] Gaspard J.C., Bauer G.B., Reep R.L., Dziuk K., Read L., Mann D.A. (2013). Detection of hydrodynamic stimuli by the Florida manatee (Trichechus manatus latirostris). J. Comp. Physiol. A.

[94] Marshall C.D., Huth G.D., Edmonds V.M., Halin D.L., Reep R.L. (1998). Prehensile Use of Perioral Bristles During Feeding and Associated Behaviors of the Florida Manatee (*Trichechus manatus latirostris*). Mar. Mammal. Sci..

[95] Sarko D.K., Reep R.L., Mazurkiewicz J.E., Rice F.L. (2007). Adaptations in the structure and innervation of follicle-sinus complexes to an aquatic environment as seen in the Florida manatee (*Trichechus manatus latirostris*). J. Comp. Neurol..

[96] Barth F.G. (2002). A Spider’s World: Senses and Behavior.

[97] Barth F.G. (1982). Geethabali Spider vibration receptors: Threshold curves of individual slits in the metatarsal lyriform organ. J. Comp. Physiol. A.

[98] French A.S., Torkkeli P.H., Ernst-August S. (2002). From stress and strain to spikes: Mechanotransduction in spider slit sensilla. J. Comp. Physiol. A Neuroethol. Sens. Neural. Behav. Physiol..

[99] Friedel T., Barth F.G. (1997). Wind-sensitive interneurones in the spider CNS (*Cupiennius salei*): Directional information processing of sensory inputs from trichobothria on the walking legs. J. Comp. Physiol. A.

[100] Barth F.G., Humphrey J.A., Wastl U., Halbritter J., Brittinger W. (1995). Dynamics of Arthropod Filiform Hairs. III. Flow Patterns Related to Air Movement Detection in a Spider (*Cupiennius salei* Keys.). Philos. Trans. R Soc. B Biol. Sci..

[101] Barth F.G., Höller A., Hilfiker S., Pieribone V.A., Czernik A.J., Kao H.-T., Augustine G.J., Greengard P. (1999). Dynamics of arthropod filiform hairs. V. The response of spider trichobothria to natural stimuli. Philos. Trans. R. Soc. B: Biol. Sci..

[102] Albert J., Friedrich O., DeChant H.-E., Barth F. (2001). Arthropod touch reception: Spider hair sensilla as rapid touch detectors. J. Comp. Physiol. A Neuroethol. Sens. Neural Behav. Physiol..

[103] Gronenberg W., Tautz J., Hölldobler B. (1993). Fast Trap Jaws and Giant Neurons in the Ant Odontomachus. Science.

[104] Just S., Gronenberg W. (1999). The control of mandible movements in the ant Odontomachus. J. Insect Physiol..

[105] Aonuma H., Osuka K., Ohkawara K. Mechanisms of Ultra-High Speed Movement in the Trap Jaw Ant. Proceedings of the 2017 56th Annual Conference of the Society of Instrument and Control Engineers of Japan (SICE).

[106] Larabee F.J., Gronenberg W., Suarez A.V. (2017). Performance, morphology and control of power-amplified mandibles in the trap-jaw ant Myrmoteras (*Hymenoptera: Formicidae*). J. Exp. Biol..

[107] Patek S.N., Baio J.E., Fisher B.L., Suarez A.V. (2006). Multifunctionality and mechanical origins: Ballistic jaw propulsion in trap-jaw ants. Proc. Natl. Acad. Sci. USA.

[108] Jaffé K., Marcuse M. (1983). Nestmate recognition and territorial behaviour in the antOdontomachus bauri emery (*Formicidae: Ponerinae*). Insectes Sociaux.

[109] Murphy C.T., Eberhardt W.C., Calhoun B.H., Mann K.A., Mann D.A. (2013). Effect of Angle on Flow-Induced Vibrations of Pinniped Vibrissae. PLoS ONE.

[110] Larabee F.J., Suarez A.V. (2015). Mandible-Powered Escape Jumps in Trap-Jaw Ants Increase Survival Rates during Predator-Prey Encounters. PLoS ONE.

[111] Mohan V., Spagna J.C. (2015). Jump Performance in Trap-Jaw Ants: Beyond Trigger Hairs. Bull. NJ Acad. Sci..

[112] Fay F.H. (1982). Ecology and Biology of the Pacific Walrus, Odobenus rosmarus divergensIlliger. N. Am. Fauna.

[113] Kastelein R.A., Stevens S., Mosterd P. (1990). The Tactile Sensitivity of the Mystacial Vibrissae of a Pacific Walrus (Odobenus rosmarus divergens). Part 2: Masking. Aquat. Mamm..

[114] Bauer G.B., Reep R.L., Marshall C.D. (2018). The Tactile Senses of Marine Mammals. Int. J. Comp. Psychol..

[115] Chapman H.C. (1875). Observations on the Structure of the Manatee. Proc. Acad. Nat. Sci. Phila..

[116] Pearson M.J., Pipe A.G., Melhuish C., Mitchinson B., Prescott T. (2007). Whiskerbot: A Robotic Active Touch System Modeled on the Rat Whisker Sensory System. Adapt. Behav..

[117] Takei K., Yu Z., Zheng M., Ota H., Takahashi T., Javey A. (2014). Highly sensitive electronic whiskers based on patterned carbon nanotube and silver nanoparticle composite films. Proc. Natl. Acad. Sci. USA.

[118] Amoli V., Kim S.Y., Kim J.S., Choi H., Koo J., Kim D.H. (2019). Biomimetics for high-performance flexible tactile sensors and advanced artificial sensory systems. J. Mater. Chem. C.

[119] Ko H., Song H., Im S., Kim H., Jang B., ShimHyungbo H., Cho D.-I., Hyoungho K., Haryong S., Seunghyun I. (2015). Bioinspired Piezoresistive Acceleration Sensor Using Artificial Filiform Sensillum Structure. Sens. Mater..

[120] Maschmann M.R., Ehlert G.J., Dickinson B.T., Phillips D.M., Ray C.W., Reich G.W., Baur J.W. (2014). Bioinspired Carbon Nanotube Fuzzy Fiber Hair Sensor for Air-Flow Detection. Adv. Mater..

[121] Brown E., Rodenberg N., Amend J., Mozeika A., Steltz E., Zakin M.R., Lipson H., Jaeger H.M. (2010). Universal robotic gripper based on the jamming of granular material. Proc. Natl. Acad. Sci. USA.

[122] Syed T.N., Jizhan L., Xin Z., Shengyi Z., Yan Y., Mohamed S.H.A., Lakhiar I.A. (2019). Seedling-lump integrated non-destructive monitoring for automatic transplanting with Intel RealSense depth camera. Artif. Intell. Agric..

[123] Zhang B., Xie Y., Zhou J., Wang K., Zhang Z. (2020). State-of-the-art robotic grippers, grasping and control strategies, as well as their applications in agricultural robots: A review. Comput. Electron. Agric..

[124] Colorado J., Barrientos A., Rossi C., Breuer K. (2012). Biomechanics of smart wings in a bat robot: Morphing wings using SMA actuators. Bioinspir. Biomim..

[125] Di Luca M., Mintchev S., Su Y., Shaw E., Breuer K. (2020). A bioinspired Separated Flow wing provides turbulence resilience and aerodynamic efficiency for miniature drones. Sci. Robot..

[126] Quist B.W., Hartmann M.J.Z. (2012). Mechanical signals at the base of a rat vibrissa: The effect of intrinsic vibrissa curvature and implications for tactile exploration. J. Neurophysiol..

[127] Quist B.W., Seghete V., Huet L.A., Murphey T.D., Hartmann M.J.Z. (2014). Modeling Forces and Moments at the Base of a Rat Vibrissa during Noncontact Whisking and Whisking against an Object. J. Neurosci..

[128] Huet L.A., Rudnicki J.W., Hartmann M.J. (2017). Tactile Sensing with Whiskers of Various Shapes: Determining the Three-Dimensional Location of Object Contact Based on Mechanical Signals at the Whisker Base. Soft Robot..

